# Establishment and application of a survival rate graph model based on biomarkers and imaging indexes after primary hepatocellular carcinoma resection

**DOI:** 10.1002/cam4.6031

**Published:** 2023-05-11

**Authors:** Yue Xu, Xiaoqin Yao, Jinmei Li, Guoyuan Zhang, Guangcheng Luo, Qiang Wang

**Affiliations:** ^1^ Department of Laboratory Medicine Affiliated Hospital of North Sichuan Medical College Nanchong People's Republic of China; ^2^ Center for Translational Medicine North Sichuan Medical College Nanchong People's Republic of China; ^3^ Faculty of Laboratory Medicine North Sichuan Medical College Nanchong People's Republic of China; ^4^ Department of Laboratory Medicine Nanchong Central Hospital Nanchong People's Republic of China

**Keywords:** biomarker, cox regression analysis, imaging index, overall survival, primary liver cancer

## Abstract

**Background:**

Primary liver cancer (PLC) is a highly malignant disease. This study developed an effective and convenient tool to evaluate survival times of patients after hepatectomy, which can provide a reference point for clinical decisions.

**Methods:**

Clinical and laboratory data of 243 patients with PLC after hepatectomy were collected. Univariate cox regression analysis, Lasso regression analysis and multivariate cox regression analysis were used to determine the best prediction index. Multivariate cox regression analysis was used to construct a survival prediction model. A receiver operating characteristic (ROC) curve, calibration curve and decision curve analysis (DCA) were used to verify the model. The patients in this model were divided into was divided into high‐risk and low‐risk groups according to the optimal cut‐off value of the ROC curve for different prognostic years. Kaplan–Meier survival analysis and log‐rank test were used to analyse the survival differences between the two groups.

**Results:**

Tumour size, portal vein thrombosis, distant metastasis, alpha‐fetoprotein and protein induced by vitamin K absence or antagonist‐II levels were independent risk factors for overall survival (OS) after PLC surgery. The area under the concentration‐time curve for 2‐, 3‐ and 4‐year survival of patients was 0.710, 0.825 and 0.919, respectively, with a good calibration of the Hosmer–Lemeshow test (*p* > 0.05) and net benefit. The mortality rates in patients with 2, 3 and 4 years of survival were 70.59%, 67.83% and 66.67% for the high‐risk group and 21.84%, 22.50% and 22.67% for the low‐risk group, respectively. The mortality rate of the high‐risk group was significantly higher than that of the low‐risk group (*p* < 0.05).

**Conclusion:**

The OS model of prognosis after PLC surgery constructed in this study can be used to predict the 2‐, 3‐ and 4‐year survival prognoses of patients, and patients with different prognosis years can be re‐stratified so that they achieve more accurate and personalised assessment, thereby providing a reference point for clinical decision‐making.

## 1 INTRODUCTION

Primary liver cancer (PLC) is the sixth most prevalent and the second deadliest cancer in the world.^1^ There were an estimated 906,000 new cases and 830,000 deaths globally in 2020.^2^ PLC is a disease with a high degree of malignancy, mainly comprising three different pathological types: hepatocellular carcinoma (HCC), intrahepatic cholangiocarcinoma (ICC) and combined hepatocellular cholangiocarcinoma (cHCC‐CCA). HCC accounts for 75%–85%, and ICC accounts for 10%–15% of all PLCs.^3^, ^4^, ^5^ In China, PLC accounts for 45.27% and 47.12% of new cancer cases and cancer‐related deaths, respectively, and the prevalence of PLC is especially high in southwest China.^6^ For high‐risk patients, the cure rate and survival time of PLC can be improved by monitoring alpha‐fetoprotein (AFP) detection, b ultrasonography, computed tomography (CT) and other examination methods.^7^ However, due to the mild clinical manifestations of PLC, surveillance compliance is poor in high‐risk patients. Therefore, the disease is diagnosed late, and the treatment effect is poor.^7^, ^8^, ^9^, ^10^, ^11^ Currently, the main treatments for PLC include surgical treatment, liver transplantation and radiofrequency ablation (RFA). Studies have shown that^12^ liver resection of one or two smaller tumours has a good prognosis. The overall survival (OS) of liver resection was similar to RFA in liver function Grade A and a single small liver cancer (less than 3 cm). However, because RFA is limited by the presence or absence of cirrhosis, in patients without cirrhosis,^13^ performing liver resection can prolong survival. Based on a large number of previous pathological data, although the OS of surgery for middle and advanced liver cancer is not satisfactory, the comprehensive treatment strategy mainly based on surgery can still benefit some patients.^3^, ^4^, ^5^

In the past few decades, a number of staging systems have been used in different regions to predict survival and provide appropriate treatment for this cancer type.^5^ For example, the staging system proposed by Okuda et al.^14^ included tumour size (size >50% or ≤50%) and clinical indicators (ascites, albumin [ALB] and total bilirubin [TBIL]). The Barcelona Clinical Classification System for Liver Cancer (BCLC) analyses tumour characteristics (size, portal vein thrombosis and presence of extrahepatic metastases), liver function, treatment and other indicators.^15^ The Italian Liver Cancer Project (CLIP) analyses lymph nodes, AFP levels, portal vein thrombosis, liver function grades and other indicators,^16^ and the Chinese University Prognostic Index (CUPI) analyses tumour‐lymph node metastasis (TNM) staging and laboratory data.^17^ Tannus et al.^18^ found that the appellate scoring system showed good correlation and CUPI had the best predictive performance. Further, Lai et al.^5^ found that except that the CUPI model was developed by improving the TNM system and combining prognostic factors for the multivariate cox model, the remaining scoring models only roughly add the sum of the scores of each variable, and their prognostic accuracy remains questionable.

In recent years, with the concept of precision medicine, accurate staging of patients, individualised treatment plans, and scientific and accurate diagnosis and treatment plans can significantly improve the prognoses of patients. In order to evaluate the prognoses of patients after hepatectomy, this study used widely discussed prognostic factors including the tumour size, number, degree of differentiation, vascular involvement, serum AFP level (elevated before surgery), serum protein induced by vitamin K absence or antagonist‐II (PIVKA‐II) level, non‐anatomical resection and portal vein thrombosis.^12^, ^19^, ^20^, ^21^ Multivariate cox regression analysis was performed to construct a prognostic model to predict the OS of PLC patients after hepatectomy and guide clinical decision‐making.

## 2 MATERIALS AND METHODS

### 2.1 Patients

**FIGURE 1:**
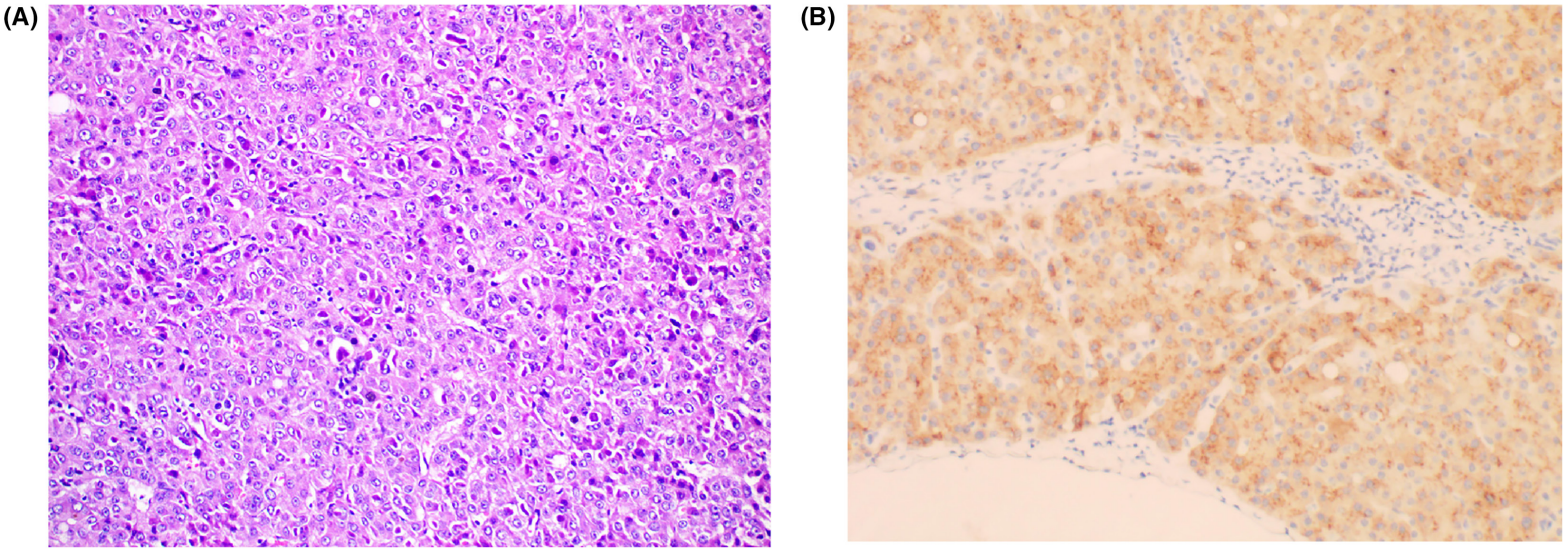
Pathological map and immunohistochemical map of primary liver cancer. (A) Pathological map. (B) Immunohistochemical map.

A total of 627 patients diagnosed with PLC after pathological biopsy were selected from our hospital from May 2016 to December 2020. The inclusion criteria were as follows: (1) All patients should undergo surgical resection under the PLC diagnosis and treatment guidelines and the guidance of professional surgeons; (2) after surgical resection of the space‐occupying lesions, the pathological diagnosis was PLC (Figure 1), with no history of other malignant tumours, and no preoperative history of other anti‐tumour treatment; (3) the first‐time treatment for the liver cancer was surgical resection; and (4) complete clinical data; and (5) the liver function score of the patient was Grade A or B and the China liver cancer staging (CNLC) stage was less than or equal to stage IIIb.^3^, ^4^, ^5^ The exclusion criteria were as follows: (1) The patient did not undergo surgical resection, and the patient's pathological diagnosis was not PLC; (2) incomplete clinical data; (3) incomplete total survival time and survival outcome; and (4) liver function score was Grade C, performance status score was 3–4, or CNLC stage was IV.^3^, ^4^, ^5^ After screening for the inclusion/exclusion criteria, a total of 243 patients were selected for follow‐ups.

### 2.2 Follow‐up

The included patients were followed‐up as outpatients or using telephone and electronic medical records, and their liver and kidney function tests and routine blood test results were reviewed every 3–7 months after treatment. The cut‐off time for survival was the time of death or survival until the end of the study, as assessed using telephone or outpatient follow‐up. The study follow‐ups were conducted until September 2022.

### 2.3 Method of treatment

Surgical excision: All patients were routinely disinfected under general anaesthesia to establish a pneumoperitoneum. Explorations of the abdominal cavity and application of laparoscopic ultrasound combined with indocyanine green fluorescence tumour imaging helped locate small lesions and mark the resection areas to obtain negative tumour margins. Tumour margins were preferentially marked at least 1 cm away from tumour boundaries. In cases where the resection margin was less than 1 cm, histological examination of the resected liver section showed no residual tumour cells or negative resection margins.

### 2.4 Method of experiment

All participants refrained from strenuous exercise or alcohol consumption for 24 h before blood collection, fasted for 12 h prior and were in a resting state before blood collection. Whenever possible, they were instructed to avoid taking intrusive medications and to note any seasonal changes. Immediately after collection, patient samples were centrifuged at 3500 rpm/min for 5 min. The lid was lifted with an automatic removing machine, and the serum or plasma was separated and introduced into the subsequent analysis device right away. PIVKA‐II was detected by a chemiluminescence particle immunoassay (Archtect 1000, Abbott); AFP by electrochemiluminescence (Cobase602, Roche, Inc.); TBIL by the vanadate oxidation method (ADVIA‐2400, Siemens); γ‐glutamyl transferase, alanine transaminase (ALT) and aspartate aminotransferase (AST) by the rate method (ADVIA‐2400, Siemens); total bile acids (TBA) by enzyme cycle assay (ADVIA‐2400, Siemens); prothrombin (PT) by the coagulation method detection technique (CP3000, Japan); platelet (PLT) and neutrophil‐to‐lymphocyte ratio (NLR) by the electrical impedance method; and ALB levels by ALB‐bromocresol green binding (ADVIA‐2400, Siemens). All ultrasound examinations were performed by qualified ultrasound practitioners in the ultrasound department of our hospital, and each examination was rechecked at least three times. MindrayM9 portable colour Doppler was used as the bedside ultrasound instrument (Shenzhen Mindray Medical Company), and 64‐slice spiral CT and magnetic resonance imaging (MRI) scans of the patients' abdomens were also performed (Achieva 1.5T, Achieva 3.0T, Siemens 3.0T, etc.). Plain scan and enhanced data were collected, and lesion features were recorded in detail. All CT and MRI scans were interpreted by two specialist physicians, and disputes were discussed until a consensus was agreed upon by both.

### 2.5 Statistical analysis

Statistical software SPSS 26.0 (IBM Corp) and R4.0.3 (R Development Core Team) were used for statistical data analysis. Kaplan–Meier survival analysis and a log‐rank test were used for univariate analysis of categorical variables. Univariate cox regression analysis was used for continuous variables. Indexes with *p* < 0.05 were included in Lasso regression to avoid overfitting. The selected indexes were included in multivariate cox regression analysis, and indexes with *p* < 0.05 were considered as independent risk factors for prognosis after PLC surgery. The survival prognosis model diagram was constructed using R language, and the reliability of the model was analysed by a receiver operating characteristic (ROC) curve, calibration degree and decision curve analysis (DCA). A value of *p* < 0.05 was considered to be statistically significant.

## 3 RESULTS

### 3.1 General data of patients and univariate and multivariate cox regression analyses

**FIGURE 2:**
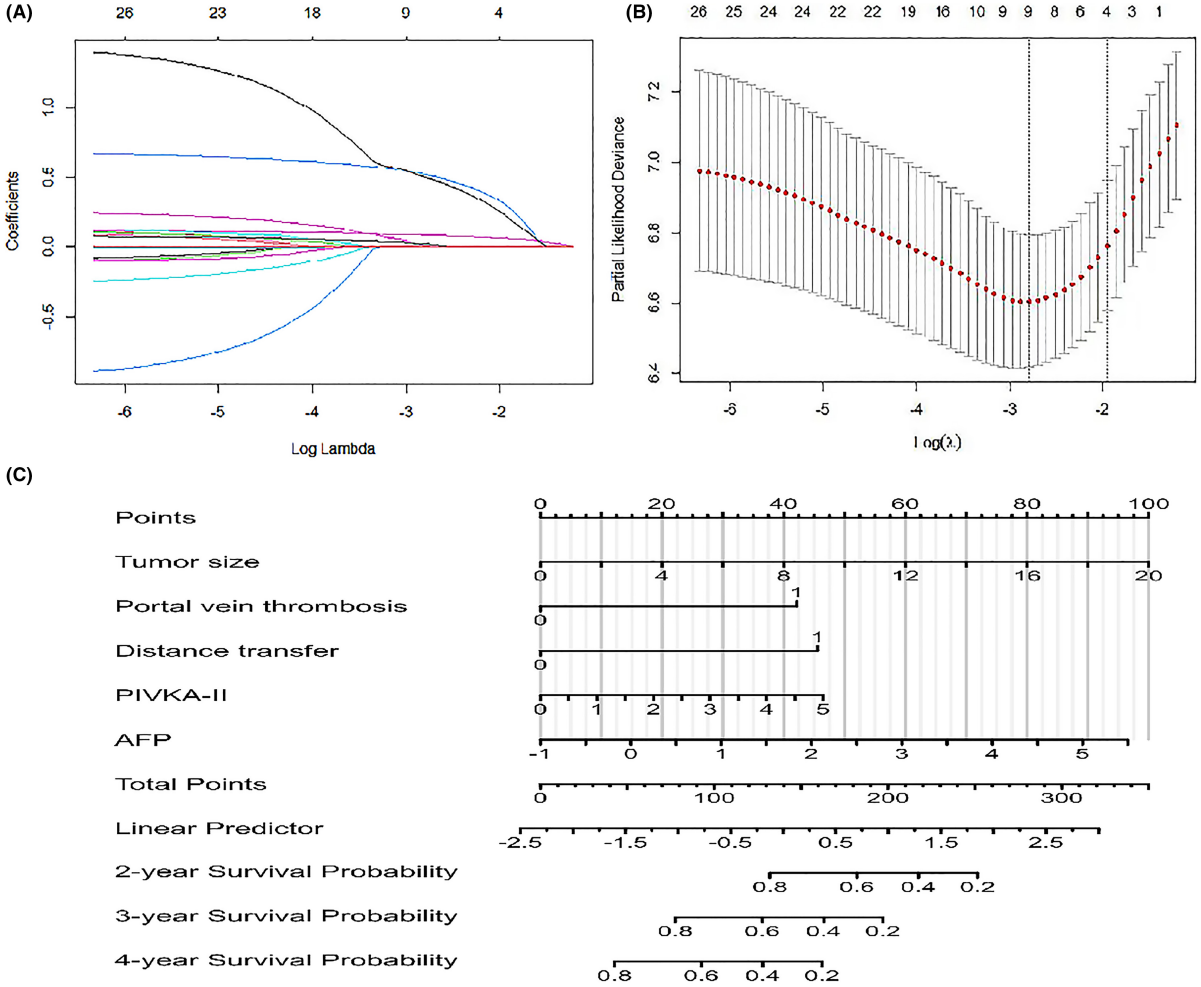
Nomogram for predicting prognosis in patients with primary liver cancer resection. (A) Lasso regression analysis. (B) 10‐fold cross‐test. (C) Nomogram.

A total of 243 patients were included in the study for construction of the survival analysis model, and the statistical description of the modelling group and the results of the single‐factor multivariate cox regression analysis are summarised in Table 1. The index age, sex, weight loss, diabetes mellitus status, number of tumours, tumour density, ALB, ALT, AST, TBA, PLT, PT and NLR ratio were excluded after applying univariate cox regression analysis (*p* > 0.05). Minimum absolute contraction and selection operator (Lasso) logistic regression (Figure 2A) and 10‐fold cross validation (Figure 2B) were applied to select the index with the highest accuracy of the model among the statistically significant indexes screened by single‐factor cox regression analysis. The results suggest that CNLC staging results, tumour boundary, tumour morphology, peripheral organ infiltration and ALP can make the model accuracy. The remaining indicators were included in the multivariate cox analysis, and it was found that tumour size, portal vein thrombosis, distant metastasis, and AFP and PIVKA‐II levels were independent risk factors for OS in patients with PLC after surgical resection (*p* < 0.05; Table 1).

**TABLE 1:** General data, univariate and multivariate analysis of the modelling group.

Variable	General Information (*n* = 243 cases)	Univariate cox analysis		Multivariate cox analysis	
		HR	*p‐*Value	HR	*p‐*Value
General clinical data					
Age	58.01 (51.00–67.00)	0.999	0.888		
Sex		1.045	0.866		
Female	29 (11.94%)				
Male	214 (88.06%)				
Weight loss		1.368	0.112		
No	191 (78.60%)				
Yes	52 (21.40%)				
Diabetes		0.651	0.182		
No	221 (90.95%)				
Yes	22 (9.1%)				
China liver cancer staging staging		1.540	0.046*		
≤IIa staging	59 (24.28%)				
≥IIb staging	184 (75.72%)				
Image‐related materials					
Number of tumours		1.067	0.707		
Single	136 (55.97%)				
Multiple	107 (44.03%)				
Tumour size	6.59 (3.50–9.00)	1.136	0.000*	1.096	0.000*
Tumour density		1.279	0.378		
Uniformity	130 (53.50%)				
Uneven	113 (46.50%)				
Tumour Boundary		1.550	0.010*		
Clear	135 (55.56%)				
Unclear	108 (44.44%)				
Tumour morphology		1.485	0.022*		
Regular	156 (64.20%)				
Irregular	87 (35.8%)				
Portal vein thrombosis		2.920	0.000*	1.835	0.003*
No	195 (80.25%)				
Yes	48 (19.75%)				
Peripheral organ invasion		1.563	0.045*		
No	208 (85.60%)				
Yes	35 (14.40%)				
Lymph node metastasis		1.876	0.001*	1.371	0.117
No	190 (78.20%)				
Yes	53 (21.80%)				
Distant transfer		2.719	0.000*	1.982	0.003*
No	72 (29.63%)				
Yes	171 (70.37%)				
Inspection‐related information					
Alpha‐fetoprotein	13,422.44 (5.80–2011.60)	1.001	0.000*	1.520	0.025*
Protein induced by vitamin K absence or antagonist‐II	6335.79 (64.52–6047.31)	1.002	0.000*	1.386	0.039*
γ‐glutamyl transferase	188.40 (54.00–247.00)	1.001	0.001*	1.000	0.479
Albumin	37.91 (34.20–42.10)	0.990	0.467		
ALP	225.70 (99.00–260.00)	1.001	0.030*		
Alanine transaminase	63.45 (25.00–70.00)	1.001	0.434		
Aspartate aminotransferase	117.38 (36–102.00)	1.001	0.077		
Platelet	78.42 (87.00–184.00)	1.000	0.939		
Prothrombin	14.31 (13.20–15.00)	1.047	0.888		
Total bile acid	19.17 (3.90–19.20)	1.004	0.125		
TBIL	25.79 (14.70–27.00)	1.009	0.000*	1.004	0.256
HBsAg		0.964	0.869		
Negative	46 (18.93%)				
Positive	197 (81.07%)				
Neutrophil‐to‐lymphocyte ratio ratio	5.51 (2.38–6.13)	1.011	0.378		

* Represents *p* < 0.05; the difference is statistically significant.

### 3.2 Establishment of prognosis survival chart

Data conversion followed this general process: natural logarithm conversion of AFP and PIVKA‐II, lg (AFP), lg (PIVKA‐II), and assigning of a value to a binary variable: no portal vein thrombosis = 0, with portal vein thrombosis = 1, without distant metastasis = 0, and with distant metastasis = 1. Independent risk factors were used to construct the OS diagram after primary HCC resection (Cox‐PLC model), and the model visualisation was constructed by drawing the diagram (Figure 2C).

### 3.3 Performance verification of Cox‐PLC model

**FIGURE 3:**
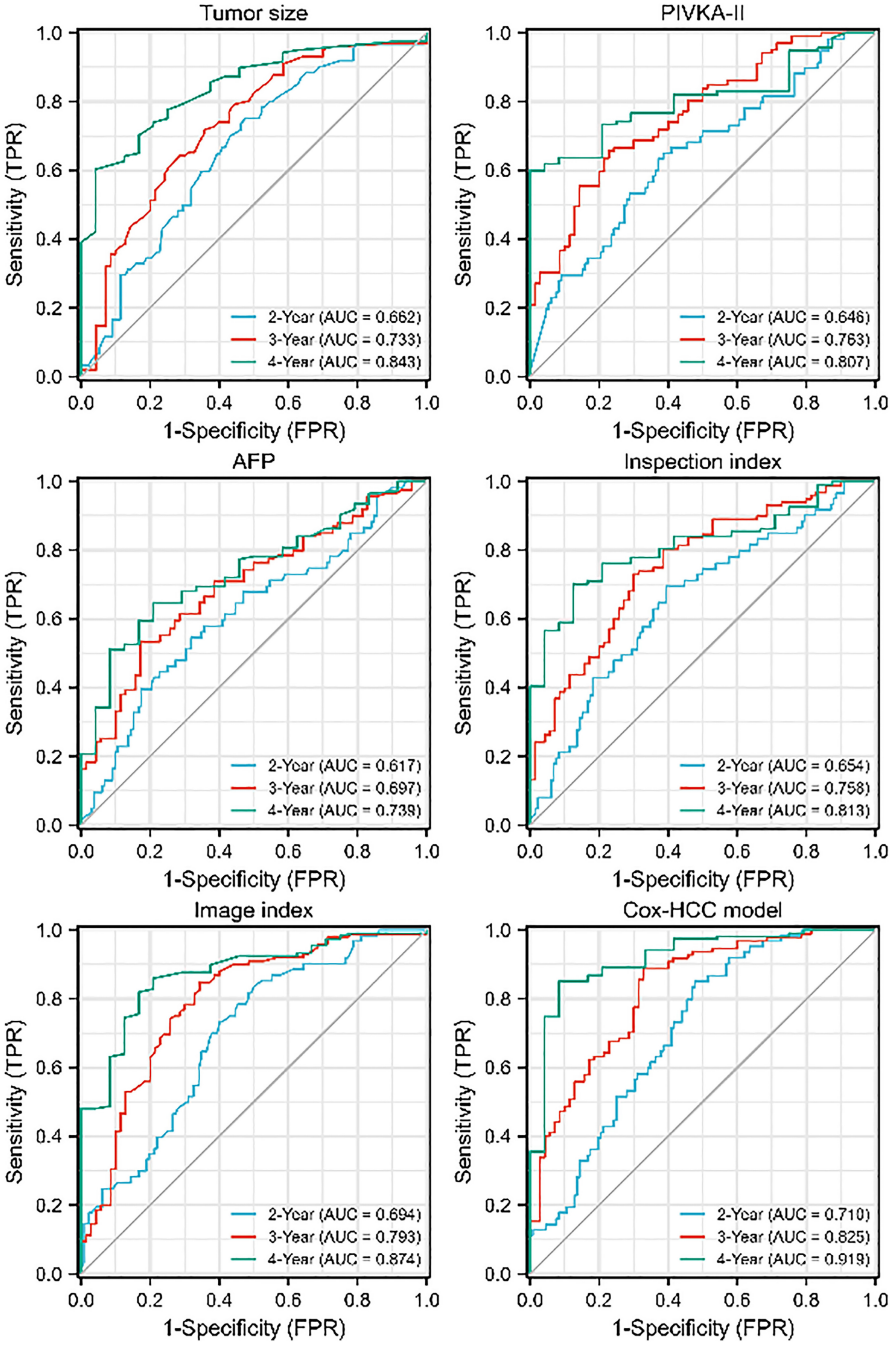
Receiver operating characteristic curves of different indexes and the Cox‐primary liver cancer model in overall survival prediction after primary hepatocellular carcinoma resection.

The joint predictor of PIVKA‐II and AFP levels was calculated by SPSS software to construct the joint index (inspection index). The combined predictors of tumour size, portal vein thrombosis and distant metastasis were calculated to construct the joint imaging index (image index). Performance indicators and comparison were implemented in different prediction models (Figure 3; Table 2).

**TABLE 2:** Area under the time‐dependent receiver operating characteristic curve for each index.

Variables	Time (year)	Area under the concentration‐time curve (95% CI)	Cut‐off	Sensitive (%)	Specificity (%)
Tumour size	2	0.662 (0.582–0.742)	4.900	75.90	52.27
	3	0.733 (0.655–0.811)	5.000	71.81	64.27
	4	0.843 (0.769–0.918)	6.000	60.32	95.77
Protein induced by vitamin K absence or antagonist‐II	2	0.646 (0.560–0.731)	2.670	65.00	61.42
	3	0.763 (0.692–0.834)	2.644	65.72	77.12
	4	0.807 (0.730–0.883)	2.604	60.02	95.45
Alpha‐fetoprotein	2	0.617 (0.530–0.705)	2.212	54.52	68.21
	3	0.697 (0.618–0.777)	2.460	53.37	82.92
	4	0.739 (0.643–0.836)	1.635	64.57	79.21
Inspection index	2	0.654 (0.571–0.738)	0.494	69.54	60.26
	3	0.758 (0.684–0.831)	0.470	73.02	70.03
	4	0.813 (0.734–0.893)	0.460	70.14	87.48
Image index	2	0.694 (0.617–0.771)	0.450	83.42	50.02
	3	0.793 (0.722–0.864)	0.442	84.84	65.67
	4	0.874 (0.805–0.943)	0.433	81.92	83.34
Cox‐primary liver cancer model	2	0.710 (0.637–0.782)	0.400	85.13	52.32
	3	0.825 (0.762–0.889)	0.370	88.78	67.10
	4	0.919 (0.858–0.970)	0.360	85.06	91.72

### 3.4 Degree of calibration

**FIGURE 4:**
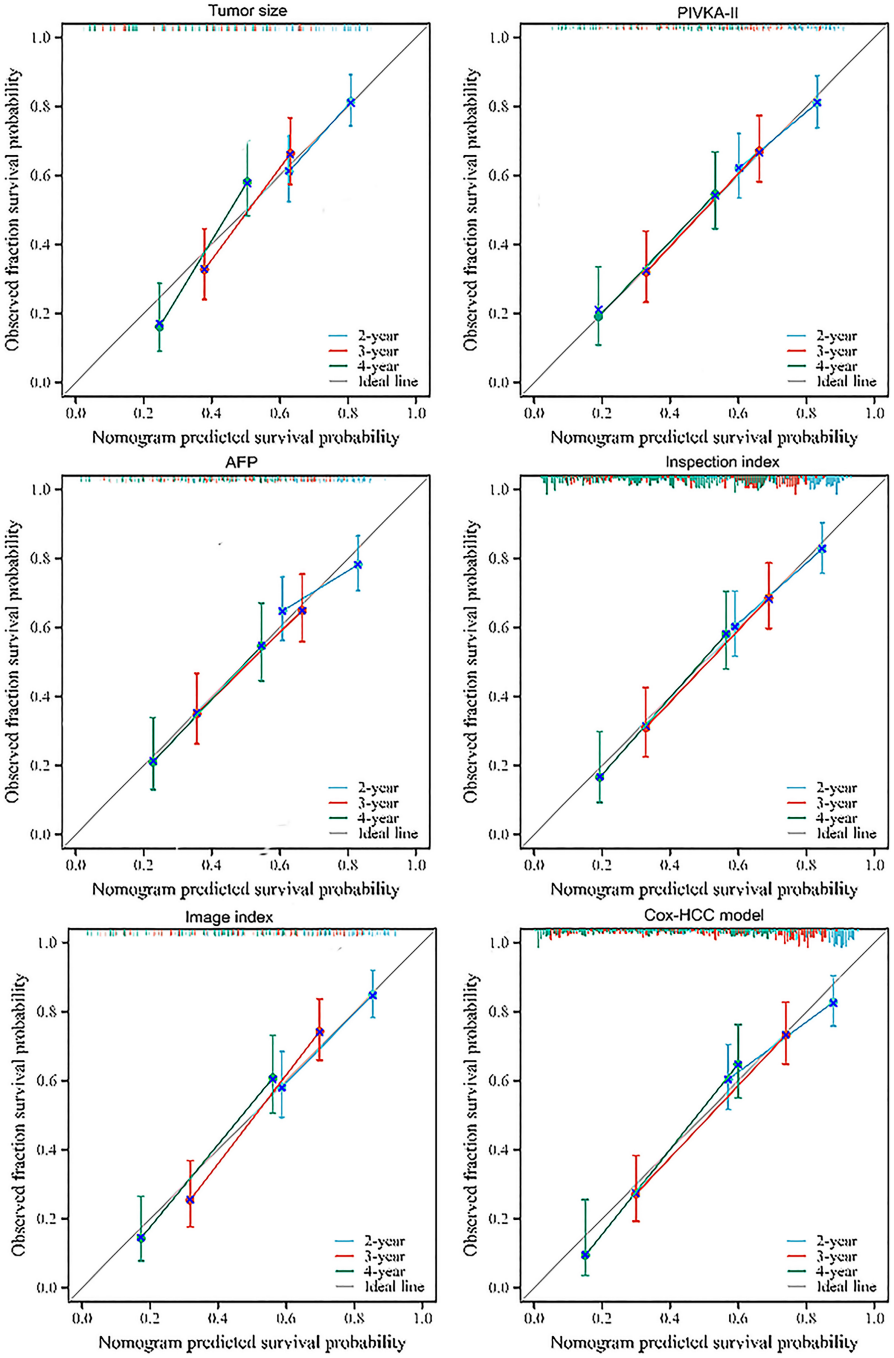
Calibration curve of different indexes and the Cox‐primary liver cancer model for predicting overall survival.

Calibration in this study was verified by a calibration curve and the Hosmer–Lemeshow test (Figure 4). The results indicated that the *p*‐values of Cox‐PLC model, tumour size, PIVKA‐II levels, AFP levels, joint index of examination and joint index of images were 0.214, 0.477, 0.277, 0.202, 0.622 and 0.819, respectively (*p* > 0.05).

### 3.5 Net benefit

**FIGURE 5:**
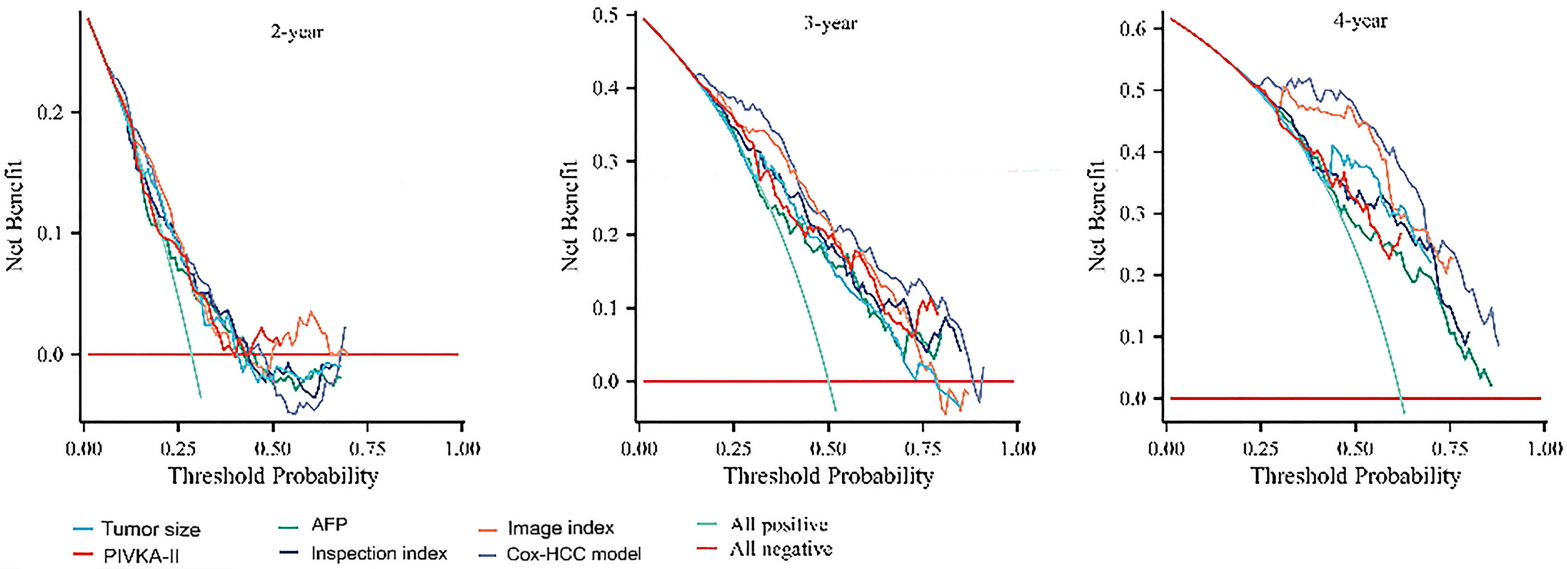
Decision curve analysis of different indexes and the Cox‐primary liver cancer model for predicting overall survival.

The net benefit of this study is mainly represented by the decision curve and the DCA curve (Figure 5). By simulating the actual clinical value of patients with or without treatment, it can be determined that the further away the case lies from the orange dotted line, the higher the clinical utility is.

### 3.6 Application of the Cox‐PLC model

**FIGURE 6:**
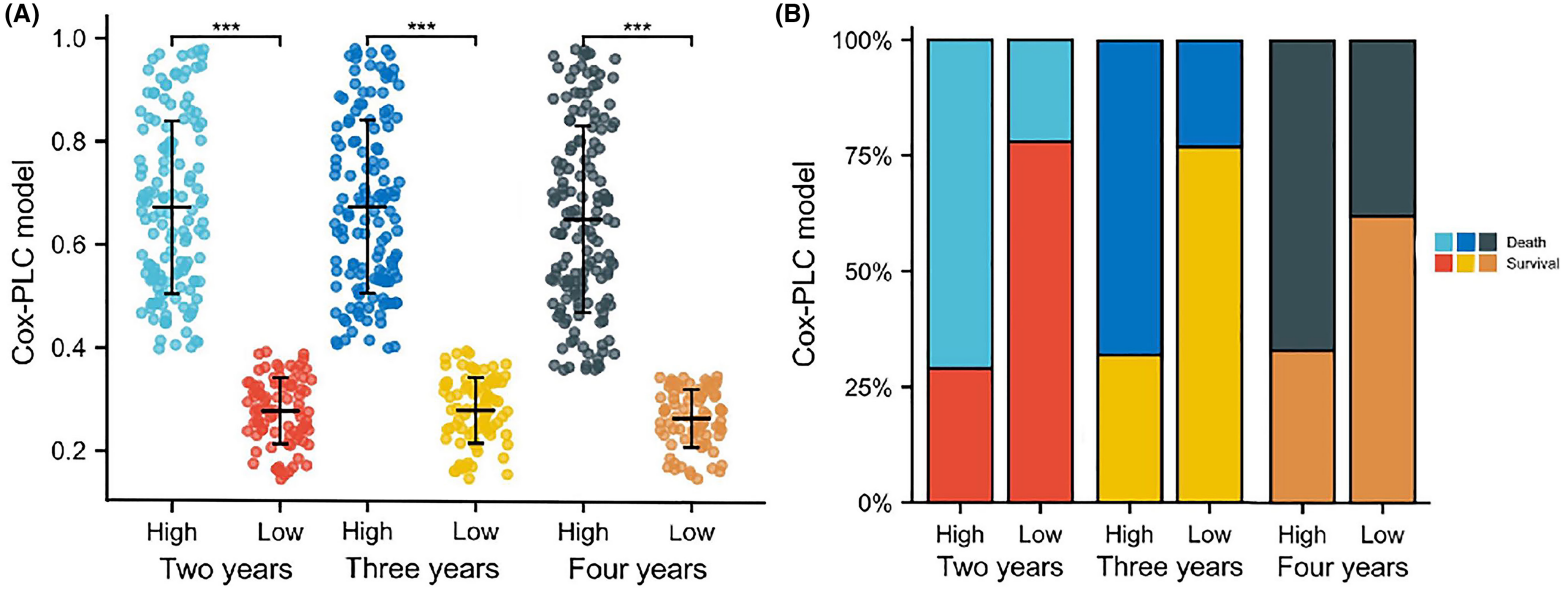
Group distribution and mortality rate of different indexes and the Cox‐primary liver cancer model. (A) Population distribution of L2‐4 and H2‐4. (B) The ratio of survival and mortality between L2‐4 and H2‐4.

Patients in the Cox‐PLC model were divided into high‐ and low‐risk groups, namely H_2‐3_ and L_2‐3_, according to the optimal cut‐off value of the time‐dependent ROC curve for 2, 3 and 4 years, respectively (Figure 6A). In the prognosis of 2, 3 and 4 years of survival in patients, the mortalities of the high‐ and low‐risk groups were 70.59%, 67.83% and 66.67% and 21.84%, 22.50% and 22.67%, respectively (Figure 6B).

**FIGURE 7:**
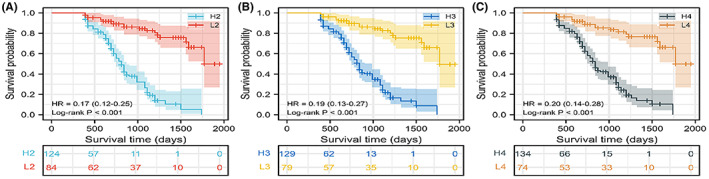
Survival analyses of patients in different risk groups. (A) Survival analyses for 2 years. (B) Survival analyses for 3 years. (C) Survival analyses for 4 years.

In the survival curves for different years, the log‐rank test results indicated that there was a statistical difference in survival time between the two groups (*p* < 0.05). (1) In the 2‐year survival prognosis, the median survival time of the high‐ and low‐risk groups was 810 (750–870) and 1770 days, respectively (Figure 7A); (2) in the 3‐year survival prognosis, the median survival time of the high‐ and low‐risk groups was 840 (750–990) and 1770 days, respectively (Figure 7B); (3) in the 4‐year survival prognosis, the median survival time of the high‐ and low‐risk groups was 840 (780–990) and 1770 days, respectively (Figure 7C).

## 4 DISCUSSION

In this study, a Cox‐PLC model for evaluating the prognosis of patients with primary HCC after resection was established by combining clinical, laboratory, and imaging indicators and by using scientific statistical methods. It was proven that its performance is better than other indexes by the time‐dependent ROC curve, calibration degree and net benefit. In the Cox‐PLC model, both mortality and median survival time increased with the degree of risk even across different prognostic years.

In this study, through univariate cox regression analysis, Lasso regression analysis and multivariate cox regression analysis, it was found that tumour size, portal vein thrombosis, distant metastasis, and AFP and PIVKA‐II levels were independent risk factors for postoperative OS in patients with PLC. The imaging features of the tumours were related to the growth pattern and growth rate of the tumour. Stress response after liver cancer surgery and portal blood flow occlusion during surgery^22^ can cause vascular endothelial injury and coagulation disorders, leading to thrombosis and invasion of the branch of the portal vein and surrounding tissues and organs, thereby affecting the prognosis of patients.^14^ AFP is an acidic protein that is synthesised by the foetal liver and inhibited after birth.^23^ AFP is the most classical serological indicator to evaluate the prognosis of liver cancer.^24^ The preoperative serum concentration is inversely proportional to the prognosis of patients.^25^ DCP, also known as PIVKA‐II, is the abnormal PT protein induced by vitamin K deficiency. In liver cells of patients with liver cancer, its concentration is increased and highly specific.^26^, ^27^ This is consistent with the independent risk factors screened in this study.

The model constructed in this study broadly incorporates clinical data, laboratory and image‐related metrics and groups patients by the optimal criticality of the time‐dependent ROC curve to re‐stratify patients at each observation time point for more accurate evaluation. In 1984, Okuda et al.^14^ first tried to combine the degree of liver disease with tumour characteristics, classifying patients according to tumour volume, and liver function data.^15^, ^28^ Compared with the present study, this study did not evaluate tumour vascular invasion and distant metastasis. These two indicators are important imaging factors for patient prognosis.^29^, ^30^ Tannus et al.^18^ found that the Okuda model lacked differentiation of survival rates of patients with early liver cancer. The CLIP model, first proposed in 1998 and internationally validated in 2000,^15^ was derived from a retrospective cohort study of 435 patients.^31^ It was followed by a prospective randomised trial that recruited 196 patients with cirrhosis and HCC^32^ and has advantages over the Okuda model in patient stratification. However, it still has the following limitations: lack of ability to identify early patients, lack of assessment of patients' overall health,^33^ lack of general applicability for hepatitis C patients^18^ and lack of good correlation with mortality.^34^, ^35^ The CUPI model was developed by a Chinese team in 2002 by improving the TNM system, combined with the prognostic factors of the multivariate cox model,^16^ and patients were divided into three stages (low risk, intermediate risk and high risk of death). The survival time of different stages of this model was significantly different, which was consistent with the results of this study. BCLC staging system^15^ was developed and published in 1999. The latest BCLC staging system consists of five stages (0, very early; A, early, B, intermediate; C, late; and D, middle to late).^36^ Stage BCLC B patient is quite broad and its recommended treatment is transarterial chemoembolization. But recently there is growing evidence that more aggressive radical treatments, such as liver resection and RFA, are feasible for selected patients with stage BCLC B HCC.^36^ In addition to being widely validated as the best and most comprehensive staging system for HCC,^37^, ^38^, ^39^, ^40^, ^41^ BCLC has also been endorsed by the European Association for the Study of the Liver^42^ and the American Association for the Study of Liver Diseases^43^ as the primary tool for defining HCC prognosis. However, Tannus et al.^18^ found in their study that BCLC could not distinguish survival time among patients in the early or middle stages of HCC, and owing to demographic differences in clinical and epidemiological parameters of HCC, the promotion and applicability of the results in other regions may be hindered. Compared with this study, except for the CUPI staging system, the appellate model only roughly adds the sum of the indicators and did not further group the population, lacking precision. Meanwhile, the model CUPI staging system uses only the Cox regression analysis. In this study, the phase regression relation number was used for weight, and the commonality between variables was avoided through Lasso analysis, which made the model more accurate and concise. In terms of model display, this study realised model visualisation through the use of the R coding language for observation of disease status.^44^ In this study, time dependence was added to the sensitivity and specificity of the time‐dependent ROC curve of the time data of individual diseases, so that the time dependence could be achieved at every time‐point. Kamarudin et al.^44^ found that the classical method of ROC curve analysis assumed that the individual time status and marker value were fixed over time; however, in practice, the disease status and marker value change over time. Therefore, it may be more appropriate to adopt a time‐dependent ROC curve. By grouping cut‐off values of observation points at different times, observation groups can be regrouped to achieve a more accurate and personalised assessment. However, this study also has some limitations. No external verification was conducted in this study, and a large number of follow‐up patients were lost during the follow‐up period. The survival time of the follow‐up patients was greater than 1 year, and the 1‐year survival prognosis was not analysed. This was a single‐centre retrospective study. The sample size can be further increased, and a multi‐centre study design can enrich and verify this model.

## 5 CONCLUSIONS

In summary, the OS model of prognosis constructed in this study can be used to predict the survival and prognosis of patients for 2, 3 and 4 years after surgical resection of PLC. Additionally, this model can be used to re‐stratify patients with different years of prognosis to allow for more accurate and personalised assessments, which will thus provide a reference for clinical decision‐making.

## AUTHOR CONTRIBUTIONS

**Yue Xu:** Conceptualization (lead); data curation (lead); formal analysis (lead); funding acquisition (lead); investigation (lead); methodology (lead); project administration (lead); resources (lead); software (lead); supervision (lead); validation (lead); visualization (lead); writing – original draft (lead); writing – review and editing (lead). **Xiaoqin Yao:** Conceptualization (equal); data curation (equal); formal analysis (equal); funding acquisition (equal); investigation (equal); methodology (equal); project administration (equal); resources (equal); software (equal); supervision (equal); validation (equal); visualization (equal); writing – original draft (equal); writing – review and editing (equal). **Jinmei Li:** Conceptualization (equal); data curation (equal); formal analysis (equal); funding acquisition (equal); investigation (equal); methodology (equal); project administration (equal); resources (equal); software (equal); supervision (equal); validation (equal); visualization (equal); writing – original draft (equal); writing – review and editing (equal). **Guangcheng Luo:** Resources (supporting); supervision (supporting). **Qiang Wang:** Resources (supporting); supervision (supporting).

## FUNDING INFORMATION

This work was supported by the Science and Technology Project of Nanchong (20SXQT0337).

## CONFLICT OF INTEREST STATEMENT

The authors confirm that they have no conflict of interest related to the data published in this manuscript.

## ETHICS STATEMENT

This study was approved by the Ethics Committee of Affiliated Hospital of North Sichuan Medical College.

## Data Availability

None.
